# Subgroup analysis of patients with HER2-negative metastatic breast cancer in the second-line setting from a phase 3, open-label, randomized study of eribulin mesilate versus capecitabine

**DOI:** 10.1007/s12282-017-0826-4

**Published:** 2018-01-04

**Authors:** Xavier Pivot, Seock Ah Im, Matthew Guo, Frederik Marmé

**Affiliations:** 1 Paul Strauss Cancer Center, 3 rue de la porte de l’hôpital , BP30042–67065 Strasbourg, France; 2 Seoul National University Hospital, Cancer Research Institute, Seoul National University College of Medicine, Seoul, Korea; 3 Oncology Business Group, Eisai Inc., Woodcliff Lake, NJ USA; 4 National Centre for Tumour Diseases, Heidelberg and Department of Gynecologic Oncology, University Hospital, Heidelberg, Germany

**Keywords:** Subgroup analysis, Eribulin, Capecitabine, Metastatic breast cancer, Overall survival

## Abstract

This post hoc subgroup analysis of a large phase 3 study compared the efficacy and safety of eribulin versus capecitabine in patients with human epidermal growth factor receptor 2 (HER2)-negative metastatic breast cancer who received second-line treatment. In the phase 3 study, women with advanced/metastatic breast cancer and ≤ 3 prior chemotherapies were randomized 1:1 to eribulin mesilate 1.4 mg/m^2^ intravenously on days 1 and 8, or twice-daily oral capecitabine 1.25 g/m^2^ on days 1–14 (21-day cycles). This analysis included 392 patients. Median overall survival was longer in patients receiving eribulin compared with capecitabine (16.1 vs 13.5 months, respectively; HR 0.77, *P* = 0.026). Median progression-free survival and response rates were similar between arms. Both treatments had manageable safety profiles.

## Introduction

The prognosis for advanced or metastatic breast cancer (MBC) remains poor, with a 5-year relative survival rate of 26% [1]. The backbone of treatment for human epidermal growth factor receptor 2 (HER2)-positive breast cancer includes anti-HER2 agents. However, for patients with HER2-negative tumors (~ 75% of patients with breast cancer) [2] who have progressed following first-line chemotherapy, no single optimal treatment has been established. Current guidelines recommend the use of sequential monotherapy to balance efficacy and toxicity [3–5]. In the second-line setting, a limited number of chemotherapeutic regimens, often administered in combinations, significantly prolonged survival [6–9]. Although single-agent capecitabine has not demonstrated a significant survival benefit after failure of chemotherapy, its safety profile makes it one of the most frequently used second-line therapies [10, 11].

Eribulin mesilate is a microtubule dynamics inhibitor with a distinct mode of action that involves binding to specific sites on the growing plus ends of microtubules [12, 13]. In the pivotal phase 3 trial (Study 305/EMBRACE), eribulin significantly improved overall survival (OS) compared with treatment of physician’s choice [14]. Study 301, which compared eribulin and capecitabine in locally advanced or MBC, showed that eribulin failed to demonstrate a statistically significant improvement in OS [hazard ratio (HR) 0.88; 95% confidence interval (CI) 0.77–1.00; *P* = 0.056] [15]. However, a separate pooled analysis from Study 301 and Study 305, requested by the European Medicines Agency (EMA), which assessed treatment effect according to HER2 status, showed that eribulin improved OS in patients with HER2-negative MBC (HR 0.82; 95% CI 0.72–0.93) [16], and specifically in the second-line or later setting (HR 0.85; 95% CI 0.76–0.94) [17]. Based on the pooled analyses, the EMA extended eribulin’s label to include routine use in a second-line setting [18]. Here, we present a post hoc subgroup analysis from Study 301 comparing the efficacy and safety of eribulin versus capecitabine as second-line treatment in patients with HER2-negative MBC.

## Methods

The study design, eligibility criteria, and statistical analyses have been described in full previously [15] and are summarized here:

### Study design summary

In the phase 3 (NCT00337103) open-label trial, patients were randomized 1:1 to receive eribulin mesilate 1.4 mg/m^2^ [equivalent to 1.23 mg/m^2^ eribulin (expressed as free base)] intravenously over 2–5 min on days 1 and 8, or capecitabine 1.25 g/m^2^ orally twice daily on days 1–14, both every 21 days, until disease progression, unacceptable toxicity, or patient/investigator request to discontinue. Patients were stratified by geographic region and HER2 status.

### Ethical approval

All patients provided written informed consent. Approval was obtained from independent ethics committees and regulatory authorities in participating countries. The study was conducted in accordance with the World Medical Association Declaration of Helsinki, guidelines of the International Conference for Harmonisation/Good Clinical Practice, and local ethical and legal requirements.

### Patient population

The primary study enrolled female patients aged ≥ 18 years with histologically or cytologically confirmed locally advanced or MBC, who had received ≤ 3 prior chemotherapy regimens (of which no more than 2 for advanced and/or metastatic disease) and prior therapy with an anthracycline and a taxane. The present analysis included patients with HER2-negative tumors (HER2-negative/hormone receptor-positive and triple negative) treated in the second line. The co-primary endpoints were OS and progression-free survival (PFS); secondary endpoints included objective response rate (ORR) and safety.

### Statistical analysis

Subgroup analysis was performed using the same statistical approaches (statistical model, missing data handling, and censoring rules) as the primary analysis. HRs of eribulin versus capecitabine were estimated in stratified Cox regression models with region as a stratification factor. *P* values of treatment differences were estimated using a Cox model. Kaplan–Meier estimates and distribution curves were determined within each arm. Safety data were summarized descriptively using data from all patients who received at least 1 dose of study treatment and had at least 1 post-baseline safety evaluation. Adverse events (AEs) were graded using Common Terminology Criteria for Adverse Events v3.0; AEs were classified using the Medical Dictionary for Regulatory Activities.

## Results

### Patient characteristics

Of the 1102 patients in Study 301, this analysis included 392 (36%) patients with HER2-negative MBC who received second-line treatment. A total of 186 patients received eribulin and 206 patients received capecitabine. Baseline patient demographics and disease characteristics were generally well balanced between treatment arms with the exception of a smaller percentage of patients with ≥ 3 organs involved (48.4 vs 56.8%, eribulin vs capecitabine, respectively) and a larger percentage of patients with non-visceral disease (16.1 vs 8.7%, eribulin vs capecitabine, respectively) in the eribulin group (Table 1).

**Table 1:** Patient demographics and baseline characteristics (second line, HER2 negative, ITT population)

Characteristic	Baseline, *n* (%)	
	Eribulin (*n* = 186)	Capecitabine (*n* = 206)
Age		
≤ 40 years	16 (8.6)	36 (17.5)
> 40 to < 65 years	135 (72.6)	150 (72.8)
≥ 65 years	35 (18.8)	20 (9.7)
Geographic region		
Eastern Europe	99 (53.2)	112 (54.4)
Latin America	39 (21.0)	37 (18.0)
Western Europe	26 (14.0)	36 (17.5)
North America	15 (8.1)	17 (8.3)
Asia	5 (2.7)	3 (1.5)
South Africa	2 (1.1)	1 (0.5)
Disease progression within 60 days after taking the last dose of taxane	81 (43.5)	118 (57.3)
ER status		
Positive	104 (55.9)	116 (56.3)
Negative	82 (44.1)	87 (42.2)
Not done	0	3 (1.5)
Hormone-receptor status		
Positive (ER- or PR-positive)	113 (60.8)	129 (62.6)
Negative (both ER- and PR-negative)	73 (39.2)	72 (35.0)
Not done	0	5 (2.4)
Triple (HER2/ER/PR)-negative	73 (39.2)	72 (35.0)
Number of organs involved		
1	37 (19.9)	27 (13.1)
2	59 (31.7)	62 (30.1)
≥ 3	90 (48.4)	117 (56.8)
Site of disease^a^		
Visceral	154 (82.8)	187 (90.8)
Non-visceral only	30 (16.1)	18 (8.7)

*ER* estrogen receptor, *HER2* human epidermal growth factor receptor 2, *ITT* intent to treat, *PR* progesterone receptor

^a^Visceral or non-visceral status was determined by independent assessment

### Efficacy

**Fig. 1:**
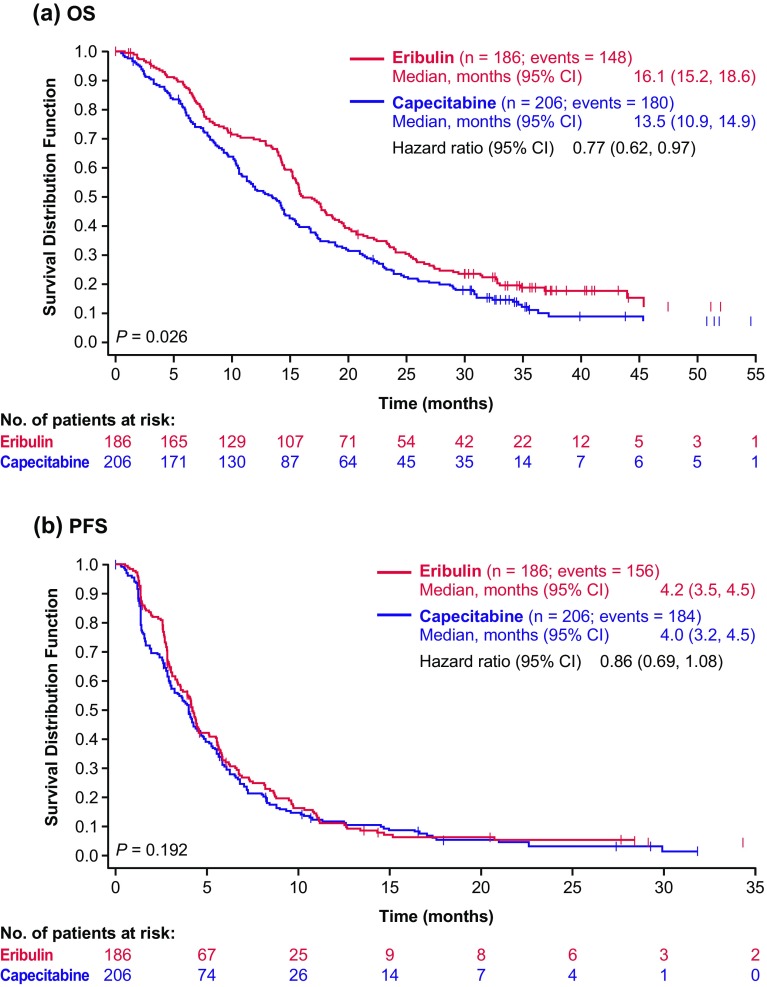
**a** Overall survival: Kaplan–Meier plot for HER2-negative patients (Study 301, second line, ITT population). **b** Progression-free survival: Kaplan–Meier plot for HER2-negative patients (Study 301, second line, ITT population).* CI* confidence interval,* HER2* human epidermal growth factor receptor 2,* ITT* intent-to-treat,* OS* overall survival, *PFS* progression-free survival

In patients with HER2-negative MBC receiving treatment in the second line, OS was longer with eribulin treatment compared with capecitabine treatment (median OS, 16.1 vs 13.5 months, respectively; HR 0.77; 95% CI 0.62–0.97; nominal *P* = 0.026) (Fig. 1a). PFS based on investigator assessment was not different with eribulin treatment compared with capecitabine treatment (median PFS, 4.2 vs 4.0 months, respectively; HR 0.86; 95% CI 0.69–1.08; nominal *P* = 0.192) (Fig. 1b). ORRs were similar between treatment arms (9.7% in the eribulin arm vs 8.7% in the capecitabine arm; *P* = 0.86).

### Safety

The most frequent treatment-emergent AEs (TEAEs) in the eribulin arm were neutropenia (53.3%), alopecia (34.8%), leukopenia (31.0%), peripheral neuropathy (pooled term, 23.9%), and anemia (21.2%) (Table 2). Grade 3 or 4 neutropenia occurred in 43.5% of patients in the eribulin arm. The most frequent TEAEs in the capecitabine arm were palmar-plantar erythrodysesthesia syndrome (48.3%), diarrhea (24.9%), nausea (21.0%), anemia (19.5%), and vomiting (19.0%). Both treatment arms had manageable toxicities consistent with their known safety profiles.

**Table 2:** TEAEs occurring at > 10% for any grade, or > 2% for grade 3 and 4

TEAEs, *n* (%)	Eribulin			Capecitabine		
	*n* = 184			*n* = 205		
	Any grade	Grade 3	Grade 4	Any grade	Grade 3	Grade 4
Patients with any TEAE	173 (94.0)	62 (33.7)	50 (27.2)	188 (91.7)	71 (34.6)	20 (9.8)
Patients with any SAE	30 (16.3)	12 (6.5)	13 (7.1)	41 (20.0)	17 (8.3)	11 (5.4)
Patients with TEAEs leading to discontinuation	15 (8.2)	8 (4.3)	2 (1.1)	18 (8.8)	6 (2.9)	4 (2.0)
Neutropenia	98 (53.3)	43 (23.4)	37 (20.1)	30 (14.6)	10 (4.9)	1 (0.5)
Alopecia	64 (34.8)	0	0	6 (2.9)	0	0
Leukopenia	57 (31.0)	17 (9.2)	3 (1.6)	19 (9.3)	2 (1.0)	1 (0.5)
Peripheral neuropathy^a^	44 (23.9)	12 (6.5)	1 (0.5)	17 (8.3)	0	0
Anemia	39 (21.2)	2 (1.1)	0	40 (19.5)	1 (0.5)	1 (0.5)
Nausea	38 (20.7)	1 (0.5)	0	43 (21.0)	2 (1.0)	0
Asthenia	36 (19.6)	10 (5.4)	0	29 (14.1)	9 (4.4)	0
Decreased appetite	29 (15.8)	0	0	32 (15.6)	2 (1.0)	0
Diarrhea	26 (14.1)	2 (1.1)	0	51 (24.9)	13 (6.3)	0
Pyrexia	26 (14.1)	1 (0.5)	0	10 (4.9)	1 (0.5)	0
Vomiting	25 (13.6)	1 (0.5)	0	39 (19.0)	4 (2.0)	0
Fatigue	25 (13.6)	2 (1.1)	0	26 (12.7)	2 (1.0)	0
Headache	24 (13.0)	1 (0.5)	0	23 (11.2)	0	0
Dyspnea	23 (12.5)	5 (2.7)	2 (1.1)	26 (12.7)	3 (1.5)	2 (1.0)
Back pain	20 (10.9)	3 (1.6)	0	16 (7.8)	1 (0.5)	0
Cough	15 (8.2)	0	0	21 (10.2)	0	0
Alanine aminotransferase increased	14 (7.6)	6 (3.3)	0	8 (3.9)	0	0
Febrile neutropenia	8 (4.3)	6 (3.3)	2 (1.1)	2 (1.0)	0	2 (1.0)
Palmar-plantar erythrodysesthesia syndrome	1 (0.5)	0	0	99 (48.3)	28 (13.7)	0

*SAE* serious adverse event, *TEAE* treatment-emergent adverse event

^a^Combines the following preferred terms: peripheral neuropathy, neuropathy peripheral, neuropathy, peripheral motor neuropathy, polyneuropathy, peripheral sensory neuropathy, peripheral sensorimotor neuropathy, demyelinating polyneuropathy, and paresthesia

## Discussion

Treatment options for patients with HER2-negative MBC remain limited, with guidelines recommending single-agent chemotherapy [3–5]. Registration of a new agent in the metastatic setting beyond the first line requires demonstration of survival benefit [19]. In Study 301, eribulin did not demonstrate a significant survival benefit compared with capecitabine. However, it did show a heterogeneous treatment effect with a more favorable OS in patients with HER2-negative disease [15, 20]. Patient populations in large randomized trials such as this one are typically heterogeneous because of worldwide accrual, with a highly variable standard of care due to the range of drugs that may be licensed in each country.

A pooled analysis from Study 305 and Study 301, requested by the EMA, demonstrated that eribulin increased survival rates compared with control treatment in patients with HER2-negative MBC and provided support for an extension of its label to include routine use in a second-line setting [16, 17]. In this post hoc subgroup analysis of Study 301, which evaluated patients with HER2-negative MBC receiving eribulin or capecitabine as second-line treatment, prolonged OS benefit with the use of eribulin versus capecitabine was observed, supporting its activity as a second-line treatment for patients with HER2-negative MBC. Disease characteristics in both treatment groups were generally similar with the exception of a smaller percentage of patients with ≥ 3 organs involved and a larger percentage of patients with non-visceral disease in the eribulin group. Both treatments had manageable, non-overlapping, safety profiles. These findings, although exploratory, may aid in treatment decisions in the absence of prospective study data in patient populations that match the approved European Union indication for eribulin. Other factors may be considered in treatment decisions for second-line therapy, including toxicity profiles, residual effects from prior chemotherapy regimens, and modality of administration.
